# Early versus Extended Exposure in Speech Perception Learning: Evidence from Switched-Dominance Bilinguals

**DOI:** 10.3390/languages5040039

**Published:** 2020-10-18

**Authors:** Michael Blasingame, Ann R. Bradlow

**Affiliations:** Department of Linguistics, Northwestern University, Evanston, IL 60208, USA

**Keywords:** heritage speakers, bilingualism, speech perception

## Abstract

Both the timing (i.e., when) and amount (i.e., how much) of language exposure affect language-learning outcomes. We compared speech recognition accuracy across three listener groups for whom the order (first versus second) and dominance (dominant versus non-dominant) of two languages, English and Spanish, varied: one group of Spanish heritage speakers (SHS; L2-English dominant; L1-Spanish non-dominant) and two groups of late onset L2 learners (L1-dominant English/Spanish learners and L1-dominant Spanish/English learners). Sentence-final word recognition accuracy in both English and Spanish was assessed across three “easy” versus “difficult” listening conditions: (1) signal-to-noise ratio (SNR; +5 dB SNR versus 0 dB SNR), (2) sentence predictability (high versus low sentence predictability), and (3) speech style (clear versus plain speech style). Overall, SHS English recognition accuracy was equivalent to that of the L1-dominant English Spanish learners, whereas SHS Spanish recognition accuracy was substantially lower than that of the L1-dominant Spanish English learners. Moreover, while SHS benefitted in both languages from the “easy” listening conditions, they were more adversely affected by (i.e., they recognized fewer words) the presence of higher noise and lower predictability in their non-dominant L1 Spanish compared to their dominant L2 English. These results identify both a benefit and limit on the influence of early exposure. Specifically, the L2-dominant heritage speakers displayed L1-like speech recognition in their dominant-L2, as well as generally better recognition in their non-dominant L1 than late onset L2 learners. Yet, subtle recognition accuracy differences between SHS and L1-dominant listeners emerged under relatively difficult communicative conditions.

## 1. Introduction

In many cases, a talker’s first language (L1) is also their dominant language. However, in many immigrant communities (including, but not limited to the USA), there is a growing number of speakers for whom their L1 is not their dominant language (Brecht and Ingold 2002). For this group, the L1 is typically spoken at home, while the L2 is generally the dominant language of the society in which the talkers live. Due to the nature of acquisition for these individuals (the L1 is acquired and used almost exclusively at home and in family-specific settings), we refer to them as *heritage speakers* (for a review, see Polinsky and Kagan 2007). For example, in the United States, there is a large Hispanic population (Ramirez and de la Cruz 1997) due to immigration from Mexico and Latin America. Within these immigrant families, children often speak Spanish at home with their parents and grandparents who have not acquired English, but must use English at school, where it is the medium of instruction and in society at large. For these children, whom we refer to as Spanish Heritage Speakers (in this study SHS), Spanish is the L1 and English is the L2 based on the age and order in which both languages were acquired. However, due to their formal education in English and their limited use of Spanish (typically restricted to home use), they often become dominant in English (Kohnert et al. 1999; Montrul 2002; Polinsky and Kagan 2007).

Since age-of-acquisition and language dominance are confounded in the typical L1-dominant bilingual individual, the “switched” dominance of L2-dominant heritage speakers provides a unique window into the separate and combined influences of early versus extended language exposure on speech and language processing. That is, comparing heritage speakers to adult L2 learners of the heritage speakers’ L1 distinguishes early versus late exposure in bilingual listeners, whose levels of knowledge of the target language appear equal, as many heritage speakers place into college-level intermediate language courses for their L1. (See Polinsky and Kagan (2007) for a review of course placement issues related to heritage speakers.) Consider Spanish Heritage Speakers (SHS) in the USA who are exposed to Spanish from birth and stop receiving continuous linguistic input in Spanish at approximately the age of five years old. The knowledge gained from this early and limited linguistic input is interrupted at five years old (Montrul 2002) and as a result, certain aspects of SHS Spanish grammar^1^ are not fully realized to dominant L1-like levels as SHS age and attend higher levels of education in English. This early, yet unsustained, exposure can then be compared to young adult learners of Spanish (approximately 18–19 years old) who receive several years of late exposure to Spanish (or similarly, English learners from other L1 backgrounds who receive later English exposure). Although the current amount of use for English and Spanish is similar in heritage speakers versus L2 learners, the manner and timeframe in which the languages were acquired are not equivalent. Despite not being symmetrical (as the exposure to Spanish for SHS continues throughout adulthood), comparing L2 learners of Spanish to heritage speakers of Spanish isolates early, yet interrupted, L1 acquisition from exclusively late L2 acquisition.

The current study focuses on L1 and L2 speech-in-noise recognition at the sentence level with a particular focus on acoustic-phonetic and semantic-contextual factors (to be defined and discussed in detail below) that are known to influence L1 and L2 speech recognition: speech style, semantic predictability, and signal-to-noise ratio (SNR). As discussed further below, previous work has shown that some sources of speech recognition enhancement are highly beneficial for L1 speech processing but provide either no benefit or less benefit for L2 speech processing. In this study, we asked which, if either, of a switched-dominance (or heritage) speaker’s two languages would show non-dominant L2-like vulnerabilities in speech recognition, the non-dominant but first-acquired L1 or the dominant but second-acquired L2. The following section outlines the various factors known to influence speech perception.

Clear speech refers to a slower, hyper-articulated speaking style that a talker uses in order to aid the listener in speech recognition (e.g., Picheny et al. 1985 and many following studies). Clear speech is associated with several acoustic correlates including a slower speech rate, increased energy in the 1–3 kHz long-term averaged speech spectrum, and changes in vowel spectra (for reviews see Krause and Braida 2004; Smiljanic and Bradlow 2009; and Smiljanic Forthcoming). Cross-language comparisons of clear speech production have shown that while many clear speech enhancements are language-general, others enhance language-specific phonological contrasts (e.g., Smiljanic and Bradlow 2005, 2008). Previous work on clear speech perception has shown that L1 listeners benefit from this speech style in adverse listening conditions compared to plain speech, a speech style that does not contain such acoustic enhancements, (e.g., Smiljanic and Bradlow 2009), as L1 listeners benefit from the enhancement of salient acoustic features (e.g., longer segments and pause durations), as well as from the enhancement of phonological contrast. Both Bradlow and Bent (2002) and Bradlow and Alexander (2007) found that L1 English listeners received a greater clear speech benefit compared to L2 English listeners on English speech-in-noise recognition tasks. Yet, other studies (e.g., Smiljanic and Bradlow 2011; Van Engen et al. 2014; Borghini and Hazan 2020) have shown that relatively high-proficiency L2 listeners can also benefit from clear speech enhancements, and that both L1 and L2 talkers are highly adaptive to listener-related communicative barriers (e.g., Hazan et al. 2012). These results suggest that clear speech production serves to enhance the speech signal in a way that facilitates speech recognition accuracy by L1 listeners, and with some experience-related modulation, L2 listeners, as well.

Semantic predictability refers to the ability of a listener to predict an upcoming word in a given utterance based on the semantic information provided by the words that preceded it. The availability of contextual information (or semantic predictability) has also been shown to affect L1 and L2 listeners differently. Specifically, L1, but not L2^2^, listeners have been shown to benefit from semantic cues in speech-in-noise processing (e.g., Mayo et al. 1997; Bradlow and Alexander 2007; Shi 2010). That is, under noisy listening conditions, when the preceding context in a sentence strongly predicted the final keyword (high semantic predictability), L1 listeners showed higher final word recognition accuracy than when the preceding context was unhelpful (low semantic predictability), yet L2 listeners received either no benefit or a reduced benefit from contextual information. This result is consistent with Shi (2010) who found that very late L2 English learners performed the worst at extracting contextual information (as evidenced by a small keyword recognition difference between high and low predictable Speech-Perception-in-Noise (SPIN) sentences taken from Bilger et al. 1984) compared to early L2 learners and monolinguals. Furthermore, L1 listeners were able to benefit from these semantic-contextual cues for speech-in-noise recognition to a much greater extent compared to any group of L2 listeners (‘early’, ‘late’ or ‘very late’), which Shi (2010) concludes is a result of semantic processing in the L2 being considerably less efficient than in the L1.

More recently, Borghini and Hazan (2020) demonstrated that L2 listeners of English were able to utilize semantic predictability and speaking style to their advantage in a speech-in-noise recognition task and, critically, that the effects of semantic predictability and speaking style were equal to the effects for L1 listeners of English. These equal results were obtained, however, by adjusting the signal-to-noise ratio to each participant’s independent threshold for noise tolerance (rather than testing for recognition accuracy at a fixed signal-to-noise ratio). The listeners in this study showed quite high recognition accuracy of speech presented under favorable (i.e., quiet) listening conditions, suggesting that they were relatively high-proficiency L2 listeners. Thus, this result suggests that semantic predictability and speaking style, while possibly attenuated for L2 listeners (as found by earlier work, e.g., (Mayo et al. 1997; Bradlow and Alexander 2007; Shi 2010)), can combine to provide robust speech recognition enhancement for both L1 and L2 listeners.

The presence of background noise is well known to lower a listener’s accuracy and to raise the effort needed to process speech effectively (e.g., Bilger et al. 1984; Paulus et al. 2020 and many others). Beyond acoustic factors that contribute to speech-in-noise processing, work in this area also highlights notable differences between L1 and L2 listeners. Mayo et al. (1997) found that age of acquisition (AoA) is a strong predictor of noise tolerance in speech-in-noise recognition tasks, suggesting that the later a listener acquires a language, the less accurately he/she will recognize speech under adverse listening conditions. However, even early L2 listeners showed speech-in-noise processing difficulties in English compared to English monolinguals. This second result is consistent with Rogers et al. 2006), who found that despite an early AoA for English, balanced Spanish-English bilinguals still showed English speech-in-noise processing deficits compared to English monolinguals. Additionally, Shi (2010) found that L1 listeners (and early AoA L2 listeners) recognized sentence-final keywords better in a speech-in-noise task at lower signal-to-noise ratios SNRs compared to late L2 listeners. Although differences in accuracy emerged even for L1 versus highly proficient L2 listeners under non-optimal listening conditions (Mayo et al. 1997; Rogers et al. 2006; Shi 2010), early L2 listeners still demonstrated better speech-in-noise recognition tasks compared to late L2 listeners, consistent with the claim that early acquisition is necessary for accurate speech-in-noise recognition (e.g., Montrul 2005).

Given these differences between L1 and L2 processing, the present study questions how signal and semantic-contextual enhancements affect L1 and L2 speech recognition for Spanish Heritage Speakers (L2-dominant) compared to late L2 learners (L1-dominant). We hypothesize that if early language exposure is sufficient for establishing dominant L1-like speech processing routines (e.g., Mayo et al. 1997; Montrul 2005), then SHS are predicted to show dominant L1-like benefits from enhancements to the speech signal in both of their languages as they have had early exposure to both English (the dominant L2) and Spanish (the non-dominant L1). However, work by Cutler et al. (1992), Kohnert et al. (1999) and Montrul (2010) stress the importance of language dominance and usage that is independent of AoA. Therefore, an alternative hypothesis is that if extended exposure and continuous usage are necessary for efficient, dominant L1-like speech processing, then SHS are predicted to behave in a more non-dominant L2-like fashion in Spanish (e.g., reduced benefits of signal enhancements, more susceptibility to noise in the signal) because, despite being their L1, they use Spanish less than their now-dominant L2 (English). This hypothesis predicts that switched-dominance bilinguals who may exhibit a balance between the two languages under optimal speech recognition conditions will still demonstrate some vulnerabilities associated with the L1, which is more typically equated to a bilingual listener’s dominant language. It is important to note that the early exposure hypothesis and the extended exposure hypothesis do not make opposing predictions for adult, L1-dominant late bilinguals acquiring a second language (i.e., the L1-Spanish and L1-English late L2 learners in the control groups of the present study). In these cases, both hypotheses predict that L1-dominant L2 learners will benefit more from signal enhancements in the dominant L1 and show less resistance against degradation in the non-dominant L2, consistent with the previous work on L1 and L2 speech processing as discussed above.

In order to test the above hypotheses, we administered a speech-in-noise recognition task in both English and Spanish for a group of Spanish Heritage Speakers (i.e., switched dominance bilinguals with Spanish as the non-dominant L1 and English as the dominant L2), as well as a group of L1-dominant English listeners learning Spanish and a group of L1-dominant Spanish listeners learning English. In this task, participants were asked to identify keywords embedded in sentence-final positions for an accuracy score in each language. Using linear mixed-effect models, we then compared the three-way interaction between listener group, signal manipulation (speech style, predictability, and signal-to-noise ratio), and task language to determine whether, if at all, the heritage speaker group patterned differently from either the L1-English/Spanish learner or L1-Spanish/English learner group. As the results will suggest, while early exposure to both languages played a crucial role in speech processing (as demonstrated by higher overall accuracy in L1 speech recognition), heritage speakers typically benefitted from signal enhancements in English, the dominant L2, while being more adversely affected by signal degradation (i.e., plain speech, low predictability, and high levels of noise) in Spanish, their non-dominant L1.

## 2. Materials and Methods

### 2.1. Participants

Participants consisted of three groups: L1-English Spanish learners (L1-E/SL), Spanish heritage speakers (SHS), and L1-Spanish English learners (L1-S/EL). Listeners in the L1-E/SL and SHS groups were explicitly required to have been placed into intermediate Spanish courses at the university level. There was no course placement requirement for L1-S/EL as the majority of this population was graduate students studying at Northwestern University. In each group, there were 12 listeners for a total of 36 listeners (L1-E/SL group: 9 female, 3 male; SHS group: 6 female, 6 male; L1-S/EL group: 2 female, 10 male). In addition to Spanish course requirements, we established criteria (explained below) to determine which group participants should be placed in based on language acquisition and use. All listeners were compensated $10 an hour for their participation. Before beginning the study, participants were asked to fill out a brief form about their demographic information and language usage. The information about the participants reported below is based on these self-reported fields. Any percentages reported are based on participant group averages. All subjects gave their informed consent for inclusion before they participated in the study. The study was conducted in accordance with the Declaration of Helsinki, and the protocol was approved by the Internal Review Board with approval IRB# STU00200566.

L1-E/SL were defined as English L1-dominant speakers learning Spanish as their L2. The average age of L1-E/SL participants was 19.9 years old (standard error = 0.46). This group was dominant in English, as the average age of acquisition (AoA) for English was between 0 and 1 years old. The average AoA of Spanish for this group was 18 years old. Participants in the L1-E/SL group were recruited from intermediate Spanish classes at Northwestern University and on average reported using English 83.5% (standard error = 6.11) and Spanish 8.3% (standard error = 1.86) of the time. As these data indicate, the L1-E/SLs were heavily English dominant.

SHS were defined as Spanish L1 speakers who grew up in a Spanish speaking home, but attended elementary school (and beyond) in the USA, where the medium of instruction was English. The average age of the SHS group was 19.7 years old (standard error = 0.36). The average AoA of Spanish for SHS was approximately 0 (birth) and ranged between 3 to 8 years old for English. Critically, SHS are English L2 dominant due to the heavy and prolonged use of English in school and the community. SHS also have fluent reading and writing skills in English based on their formal education in the USA in which the medium of instruction is English, but not necessarily in Spanish as evidenced by SHS participants being placed in intermediate level Spanish courses at Northwestern University on the basis of a Spanish language placement test. Further evidence that SHS are English dominant comes from their self-reported values of language use in both English and Spanish. SHS indicated on average that they used English 74.45% of the time (standard error = 5.1), while reporting that they use Spanish on average 21.58% of the time (standard error = 2.84).

The L1-S/ELs were defined as Spanish L1-dominant speakers, who grew up in Latin America and attended high school where the medium of instruction was Spanish. The average age of L1-S/EL was 24.8 years old (standard error = 1.17). The average AoA of Spanish for L1-S/EL was approximately 0 (birth) and the average AoA for English was 11 years old. Evidence that L1-S/EL were clearly Spanish dominant comes from self-reported use of English and Spanish. L1-S/EL reported using English 32.3% of the time (standard error = 6.21), while Spanish use was 55% of the time (standard error = 6.64). All of the L1-S/EL speakers were graduate students at Northwestern University, so it is unsurprising that they would self-report a higher English use (their non-dominant language) compared to the SHS and L1-E/SL Spanish use at the time of testing.

Additionally, the contexts in which Spanish and English are used differed across participants. We examined the self-reported contexts in which each listener group used Spanish and English. We found that whereas L1-E/SL and L1-S/EL generally had L2 usage restricted to classroom and colleague settings, SHS reported using Spanish primarily with parents, siblings, friends, and other relatives. These SHS language patterns are consistent with Polinsky and Kagan (2007), who have shown that a defining feature of SHS is that they generally restrict their Spanish use to close friends and family.

**Figure 1.**
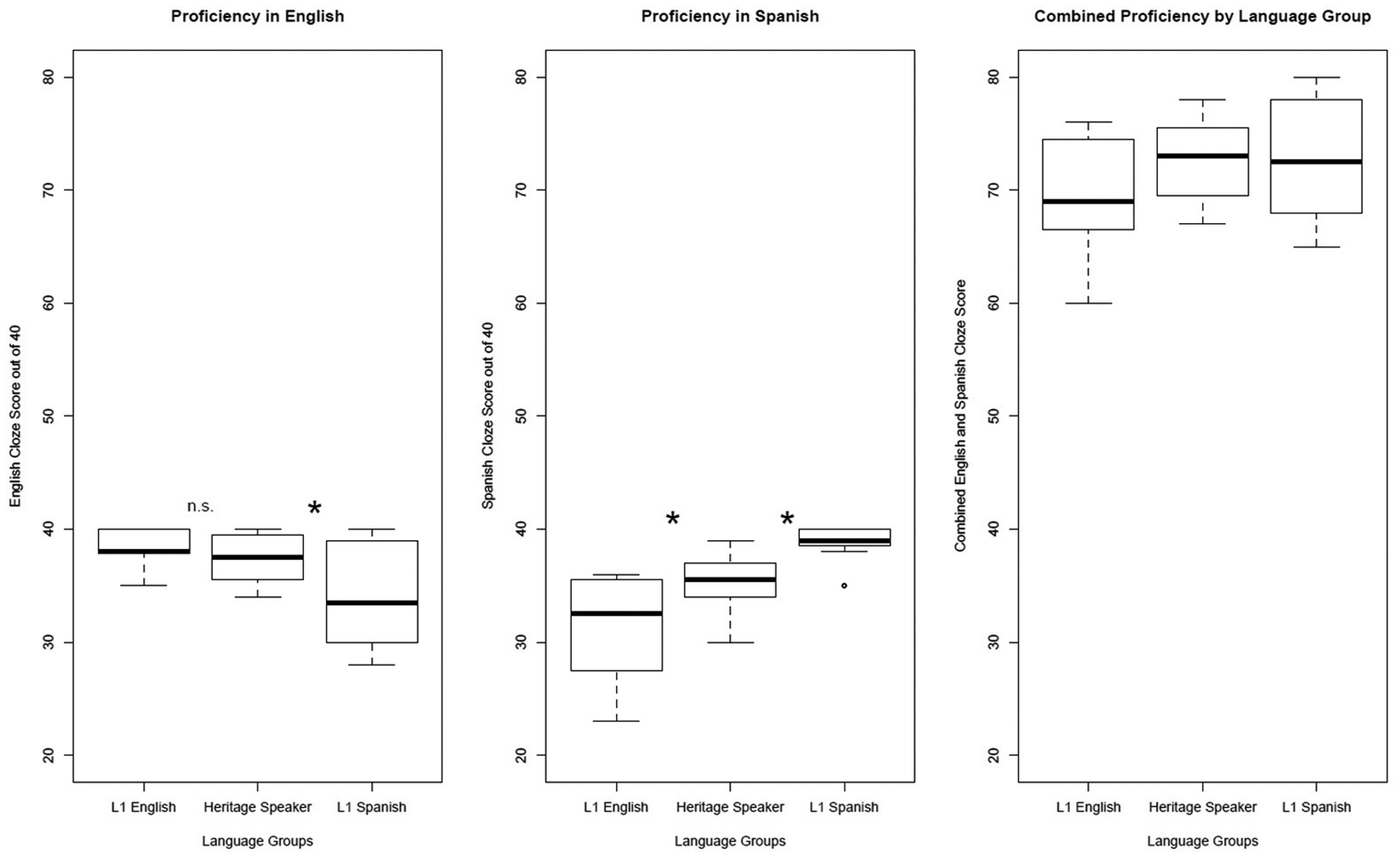
Proficiency for all listener groups for English (**left**), Spanish (**middle**), and for both languages combined (**right**).

Proficiency in both languages for all participants was assessed with a written cloze test^3^, which was given to each participant in both English and Spanish after speech recognition testing. The cloze test consisted of a passage with 40 blanks. Participants were instructed to fill in each blank as best they could to appropriately complete each sentence. The blanks in the cloze tests are designed to target specific syntactic (including inflectional and derivational morphology), semantic, and pragmatic features in the language of the test. The cloze tests are not a measure of oral proficiency, but rather a measure of explicit grammatical knowledge in written Spanish and English. Spanish Heritage speakers (SHS) had significantly higher cloze proficiency than L1-E/SL in Spanish (35.5 vs. 32.5, Bonferroni-corrected unpaired *t*-tests, t(22) = 2.55, *p* = 0.04), and there were no significant differences between L1-E/SL and SHS in English proficiency (38 vs. 37.5, Bonferroni-corrected unpaired *t*-tests, t(22) = −1.13, *p* > 0.05). Comparing L1 Spanish speaking L1-S/EL to SHS, we also found significantly lower SHS Spanish cloze proficiency (39 vs. 35.5, Bonferroni-corrected unpaired *t*-tests, t(22) = −4.1, *p* < 0.01) but higher SHS English cloze proficiency (37.5 vs. 33.5, Bonferroni-corrected unpaired *t*-tests, t(22) = 2.36, *p* < 0.05). It is important to note that the SHS combined language proficiency (English plus Spanish proficiency = 37.5+35.5 = 73) substantially exceeds that of their L1-E/SL counterparts (English plus Spanish proficiency = 38 + 32.5 = 70.5) and is equivalent to that of the L1-S/EL counterparts (English plus Spanish proficiency = 33.5 + 39 = 72.5). The results of written proficiency in English and Spanish for all groups are shown in Figure 1.

### 2.2. Materials

The materials for this study consisted of specially designed Spanish and English sentences each with a single keyword (60 keywords in Spanish and 60 keywords in English) in sentence-final position and the rest of the sentence (preceding the final keyword) serving as the context. These sentences were presented to listeners in conditions that varied by use of three factors: speech style of the talker, predictability of the target word, and signal-to-noise ratio (SNR).

Speech style refers to the difference between “clear” speech and “plain” speech. Clear speech is a speech mode that talkers adopt to increase intelligibility for listeners via acoustic enhancements such as vowel space expansion and speech rate reduction. It has been shown that clear speech produces a speech-in-noise recognition benefit over plain speech^4^ (for review see Smiljanic and Bradlow 2009). Furthermore, as L1 and L2 listeners do not equally benefit from clear speech (Bradlow and Bent 2002; Bradlow and Alexander 2007), we expected each group to receive different benefits of clear speech based on the task language (English or Spanish).

The second factor, predictability, refers to the contribution of the non-final words in a given sentence to recognition of the final keyword. Sentence frames that were “high predictability” were normed (to be discussed below) such that the final keyword could be correctly guessed based on the preceding words. Sentence frames that were ‘low predictability’ contained no such contextual cues to the final word. All English stimuli were taken from Bradlow and Alexander (2007), having been previously normed on L2 speakers of English. An example of an English keyword in both high and low predictability frames is given below; the keyword is in **bold**:

1. High versus Low Contextual Predictability
  - High contextual predictability: The meat from a pig is called **pork**.
  - Low contextual predictability: Father thought about the **pork**.

The current study normed new Spanish stimuli (see below) on L2 Spanish learners following a similar protocol used in Bradlow and Alexander (2007). Because of the high and low predictability frames for each keyword, the current study had a total of 120 sentences per language (60 keywords with two predictability frames each).

The third factor was signal-to-noise ratio (SNR), set at +5 dB (high) and 0 dB SNR (low) for each language. Studies, such as Shi (2010) and Mayo et al. (1997), have found that higher levels of noise (that is, poorer SNRs) differentially reduce speech-in-noise recognition for bilingual listeners with various ages of L2 acquisition (AoA).

#### 2.2.1. Stimuli Norming

Predictability for Spanish keywords was measured in a similar way to Bradlow and Alexander (2007). To obtain highly predictable sentences, we first obtained norms from a list of 150 highly predictable sentence frames in Spanish from Cervera and González-Alvarez (2010). Six participants from intermediate level Spanish courses at Northwestern University were recruited to participate in the first phase of norming. Participants were paid $10 an hour for their participation. Sentences were presented on a screen with the last word blanked out, and participants were instructed to fill in the last word of every sentence to the best of their abilities. As expected, since these sentences were normed on L1 Spanish speakers in Spain, the six participants in the current phase showed less than 20% agreement across stimuli. That is, only 20% of keywords had at least 4/6 participants converging on the same keyword. However, the lexical items obtained from their output were used to create new sentence frames based on vocabulary that was more accessible to intermediate learners of Spanish.

Phase two consisted of eight new paid participants, all intermediate level learners of Spanish at Northwestern University. Participants in this phase were presented with 100 sentences in which the final word was highly predictable from the context that preceded it, but this time with vocabulary that was reflected in Phase One’s results. Results contained 67 sentences with at least 88% (7 out of 8 participants) agreement on final word response, indicating that the context from early words in the sentence strongly predicted the final keyword.

In phase 3, the top 70 sentences (67 sentence frames with at least 88% agreement and 3 with at least 75% agreement) were selected for additional testing on 12 new intermediate level Spanish learners at Northwestern University. However, during this phase, both grammatical gender forms of the article (*the* or *a*) were given in Spanish to participants so as to eliminate any influence of grammatical gender on predictability. For example, in Spanish the word “the” can be written as either *el* or *la*, indicating that the morphological gender of the noun that follows is either masculine or feminine, respectively. To eliminate any artificial effects of gender influencing predictability, these 12 participants saw sentences with *el*/*la* (or *la*/*el*) before each final word when necessary. The ordering of these articles was randomized across all 70 sentences.

1. Norming Stimuli in Spanish
  - No te miento, digo el/la _________.
    ‘I’m not lying; I’m telling the _______.’
  - A Susana le gusta eschuchar la/el ________.
    ‘Susan likes listening to the _______.’

We selected the best 60 sentence frames for the current study’s stimuli. No sentences contained the same keyword across languages (e.g., if *dark* was a keyword in English, its Spanish equivalent *oscuro* was not). Additionally, the stimuli contained no cognates across languages in order to avoid any confounds based on lexical similarity across languages. Appendix A contains a full list of high and low predictability sentences in both Spanish and English.

#### 2.2.2. Word Familiarity

In order to confirm that participants in the norming study understood all content words in the stimuli, a word familiarity-rating test was administered to each participant after stimuli norming. All content words from the 100 stimuli in the second phase were presented on paper. Content words that had inflections were presented in dictionary form (verbs, infinitive ending; adjectives, in singular, masculine ending). A rating of 1 indicated “I am unfamiliar with this word and I do not know how to use it in Spanish”. A rating of 4 indicated “I recognize this as a Spanish word, but I am unsure of its meaning”. A rating of 7 indicated “I recognize this as a Spanish word and I know how to use it”. Participants were asked to provide a rating from 1–7 and provide an English translation if possible. The median rating for all content words used in the study was 6.92 (out of 7) with a standard deviation of 0.49, indicating that participants were highly familiar with all content words in the final stimuli and that vocabulary level was unlikely to influence agreement of the final keywords. Two additional L1 Spanish speakers checked all of the final 60 stimuli for grammar and spelling errors.

#### 2.2.3. Stimuli Recording

Stimuli were recorded in a sound-attenuated booth using a Shure SM81 Condenser Handheld microphone. The talker for the stimuli was a simultaneous Spanish-English bilingual for whom the age of acquisition of both languages was approximately 0 (at birth). Sentences were displayed on a computer monitor one at a time with breaks every 20 sentences to avoid fatigue and disfluencies. Speech style and language were blocked such that Spanish was recorded first, followed by English. Clear speech always preceded plain speech styles to avoid any second-mention reduction effects (e.g., Baker and Bradlow 2007) in the clear speech stimuli. To elicit clear speech stimuli, the talker was instructed to speak slowly and clearly, as if she were talking to someone who was having difficulty understanding her. To elicit plain speech stimuli, the talker was instructed to speak comfortably as if she were talking to someone who was familiar with her speech patterns. Sentences were trimmed to remove silences on both ends and leveled to the same overall RMS amplitude in Praat (Boersma and Weenink 2020).

#### 2.2.4. Accent Rating

Three L1 Spanish and three L1 English speakers were asked to rate the stimuli recorded by this simultaneous bilingual talker. 20 sentences were randomly selected from each language and given to raters who were instructed to rate her accent on a scale from 1 to 7. 1 indicated “this talker has an L2 accent/is a highly accented speaker of the language” and 7 indicated, “this talker has an L1 accent/is an unaccented speaker of the language”. The average rating for English was 6.75/7 and for Spanish 6.3/7, indicating that this talker is a highly proficient, unaccented speaker of both English and Spanish.

### 2.3. Procedure

The experiment took place over two days to avoid confounds of language mixing. Day 1 was entirely in English and Day 2 was entirely in Spanish. Because the heritage speakers (the group of interest) were English dominant, we expected that the Spanish session would be the more difficult experimental session. Therefore, SHS would benefit from the Spanish session coming second as they would be able to accommodate to the task, effectively making it harder for us to observe lower cores in Spanish. Any lower abilities to recognize Spanish than English speech-in-noise would then not be able to be accounted for solely on the basis of a lack of task adaptation.

The study is a fully crossed design with three factors at two levels (enhancement/high or degradation/low) each. As discussed above, the three factors included signal-to-noise ratio (SNR), semantic predictability, and speech style. SNR was set at +5 dB (enhancement/high) and 0 dB (degradation/low) for both languages. The noise used in the study was speech-shaped noise leveled at the respective SNRs (+5, 0 dB) by leveling all sentences to the same baseline and then manipulating the level of the speech-shaped noise to generate both +5 and 0 dB SNR differences. To reduce perceptual difficulties due to the onset of noise and speech occurring at the same time, each sentence was surrounded by a 500 ms noise lead or lag at the start or end of the stimulus, respectively. Semantic predictability consisted of 60 highly predictable frames (enhancement/high) in English and Spanish and 60 frames in English and Spanish that did not contribute to the context of the sentence (degradation/low). Speech style was either clear speech (enhancement/high) or plain speech (degradation/low).

Stimuli were blocked by SNR with the easier +5 dB SNR coming first for both languages. This ensured that any observed reduction in final word recognition accuracy in the harder SNR (0 dB) would have exceeded any benefit gained from a task practice effect. Speech style (clear versus plain) and predictability (high versus low) were randomized within each block of SNR to avoid task learning confounds. If speech style were blocked, participants could be more likely to pay less attention to the content of the sentence during the clear speech block, since they would have realized that the final word was always going to be produced in a clear speaking style. Additionally, the low predictability sentence frames often repeat elements such as “Mom read about the … ” or “Dad thought about the … ”. By completely randomizing predictability, participants had less opportunity to learn that a particular group of keywords would not be identifiable based on context and thus, they were forced to listen to the entire sentence each time.

The same participant heard each keyword only twice: once in a high predictable context and once in a low predictable context within the same SNR block. SNR and speech style were held fixed for each keyword by participant. For example, a given keyword might be presented at +5 dB SNR and in clear speech in both the high and low predictability contexts. Thus, the effect of predictability could be assessed as a within-participant factor with SNR and style fixed.

Using MaxMSP software for stimulus presentation, participants listened to each sentence one at a time over Sony MDRV700 headphones in a sound attenuated booth. Participants were instructed to type in the last word of every sentence and that they could guess or type, “I don’t know” if they were unsure of the final word.

In order to score the outputs, participants’ responses were matched with the actual keyword. Correct identification of the final keyword received a score of 1, while an incorrect score received a score of 0. Spelling errors were ignored, specifically in cases where incorrect spelling did not contribute to a difference in meaning (e.g., in Spanish, the letters b and v are realized in all phonological contexts as the same phoneme /b/ and are considered equally correct). That is, if a participant misspelled or mis-inflected (specifically, Spanish gender) a word in a way that indicated accurate auditory recognition, the word was still counted as correct receiving a score of 1. Incorrect number markings (i.e., plural markings) were considered incorrect in both languages. In the case of multiple inflectional errors such as gender and number in Spanish, the word was then considered incorrect receiving a score of 0.

## 3. Results

The main goal of this study is to examine differences in L1 and L2 speech processing between two types of bilinguals: L2 learners (L1-dominant) and heritage speakers (switched-dominance, L2 dominant bilinguals). As such, we built two separate logistic mixed effects models comparing the SHS group to each of the two L1-dominant learner groups: (1) SHS versus L1-English/Spanish learners (L1-E/SL), and (2) SHS versus L1-Spanish/English learners (L1-S/EL). The structures for these two models were identical. Both models included each of the three enhancement factors (speech style, predictability, and signal-to-noise ratio (SNR)) as fixed factors. The interactions between all factors (e.g., speech style by predictability) were also included. All factors were contrast coded to their respective enhancement/high and degradation/low values. Language of the task (English versus Spanish) and listener group (L1-E/SL versus SHS, and L1-S/EL versus SHS) were also contrast coded within each model to compare the effects of each language on the three listener groups. As the interactions between language, listener group, and each signal manipulation are the critical comparisons for this study, these three three-way interactions were also included in both models for assessment (e.g., compare the effect of speech style in the Spanish stimuli for the SHS group versus for the L1-E/SL group). The maximal random effects structure that converged was utilized for both models. The final two models included random intercepts for participant and keyword, and random slopes included all main effects (except listener group) and a language-factor interaction for each signal manipulation by subject and predictability by keyword. The significance of each factor (and interaction) was then determined within each model using the model comparison method, which relies on chi-squared values to determine whether the presence of a single (n − 1) factor (or interaction) significantly improves the fit of the data. The results of both models, which separately compare SHS to each of the two L1-dominant/learner groups, will be presented together, grouped by each enhancement factor (speech style, semantic predictability, and SNR).

### 3.1. Speech Style

**Figure 2.**
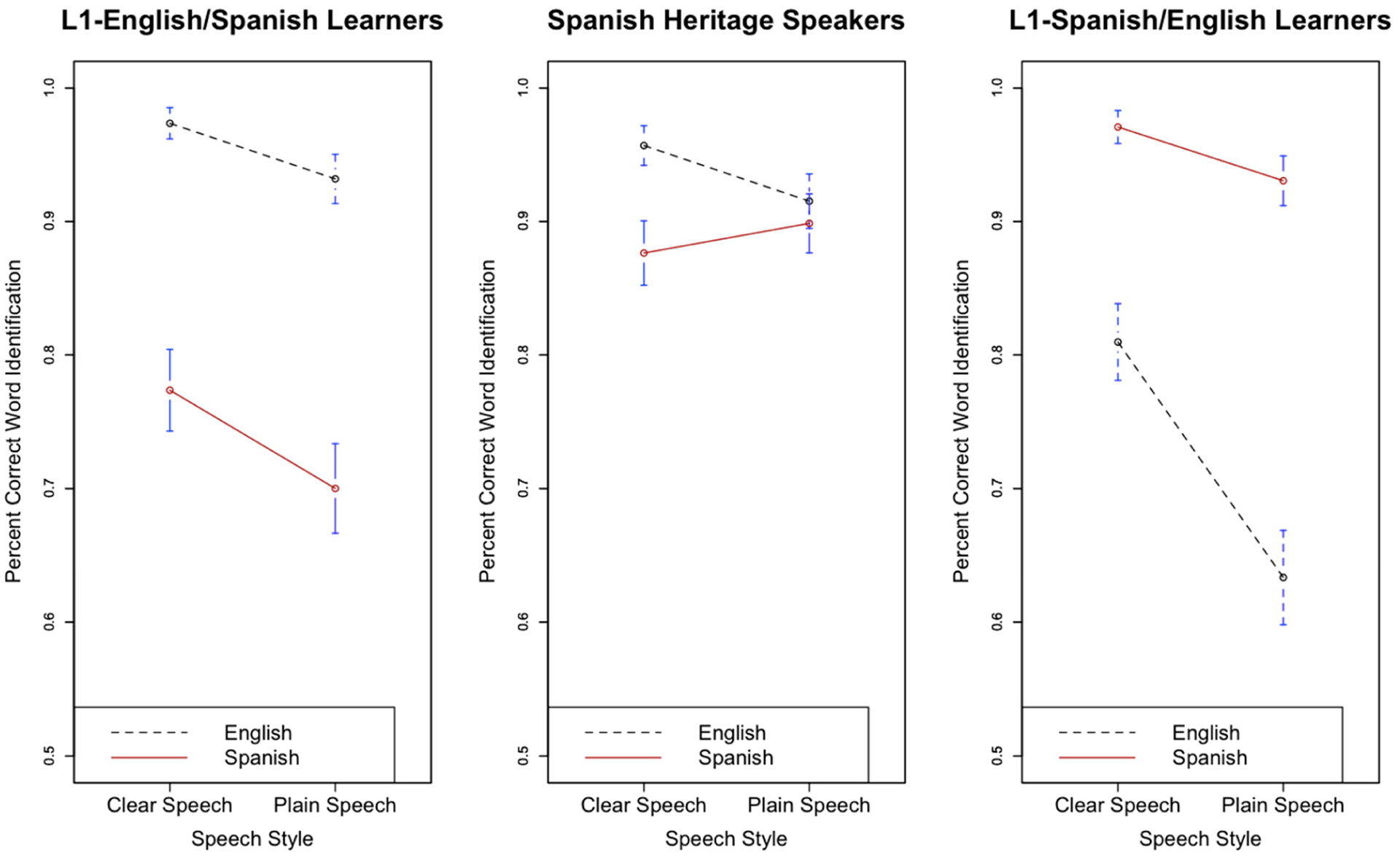
Effect of speech style on final word recognition in English and Spanish for L1-English/Spanish (L2) Learners (**left**), Spanish Heritage Speakers (**middle**), and L1-Spanish/English (L2) Learners (**right**). Bars indicate 95% confidence intervals around the means.

Figure 2 displays the effect of speech style (clear versus plain) on English and Spanish speech-in-noise recognition for all three listener groups.

With respect to L1-E/SL versus SHS, there was a significant main effect of speech style (β = 0.79, s.e. β = 0.1, χ^2^(1) = 24.57, *p* < 0.05), indicating that words produced in clear speech were more likely to be correctly recognized than those produced in plain speech. The interaction between style and language was also significant (β = 0.78, s.e. β = 0.17, χ^2^(1) = 14.01, *p* < 0.05), which suggests both L1-E/SL and SHS benefitted more from English clear speech than Spanish clear speech. This result is consistent with Bradlow and Bent (2002), who showed that bilingual listeners generally benefit more from clear speech in their first (or in this case, dominant) language than from clear speech in their second (or in this case, non-dominant) language. The two-way interaction between listener group and style was also significant (β = −0.45, s.e. β = 0.26, χ^2^(1) = 4.52, *p* < 0.05) such that SHS showed less of an overall benefit in clear speech compared to L1-E/SL. However, the three-way interaction between listener group (L1-E/SL versus SHS), language, and style was not significant (β = 0.467, s.e. β = 0.477, χ^2^(1) = 1.7, *p* > 0.05), suggesting that neither of these two-way interactions were influenced by the remaining factor.

With respect to L1-S/EL versus SHS, there was a significant main effect of speech style (β = 1.05, s.e. β = 0.11, χ^2^(1) = 23.43, *p* < 0.05), indicating that keywords produced with clear speech were more accurately recognized than keywords produced with plain speech. The three-way interaction between style, language, and listener group was significant (β = 1.33, s.e. β = 0.5, χ^2^(1) = 6.33, *p* < 0.05), suggesting that SHS and L1-S/EL did not equally benefit from speech style across both languages. That is, SHS failed to benefit from clear speech in Spanish, whereas L1-S/EL benefitted from clear speech in both languages. Additionally, there was a significant interaction between style and listener group (β = −0.92, s.e. β = 0.24, χ^2^(1) = 7.89, *p* < 0.05), which, like above, suggests that SHS showed an overall smaller benefit from clear speech. The interaction between style and language was also significant (β = 0.81, s.e. β = 0.19, χ^2^(1) = 12.45, *p* < 0.05) such that the overall benefit for clear speech was greater in English than in Spanish. While this result may be inconsistent with Bradlow and Bent (2002), it is consistent with other studies (e.g., Smiljanic and Bradlow 2011; Borghini and Hazan 2020) which also showed some benefit of clear speech for relatively high-proficiency L2 listeners.

### 3.2. Semantic Predictability

**Figure 3.**
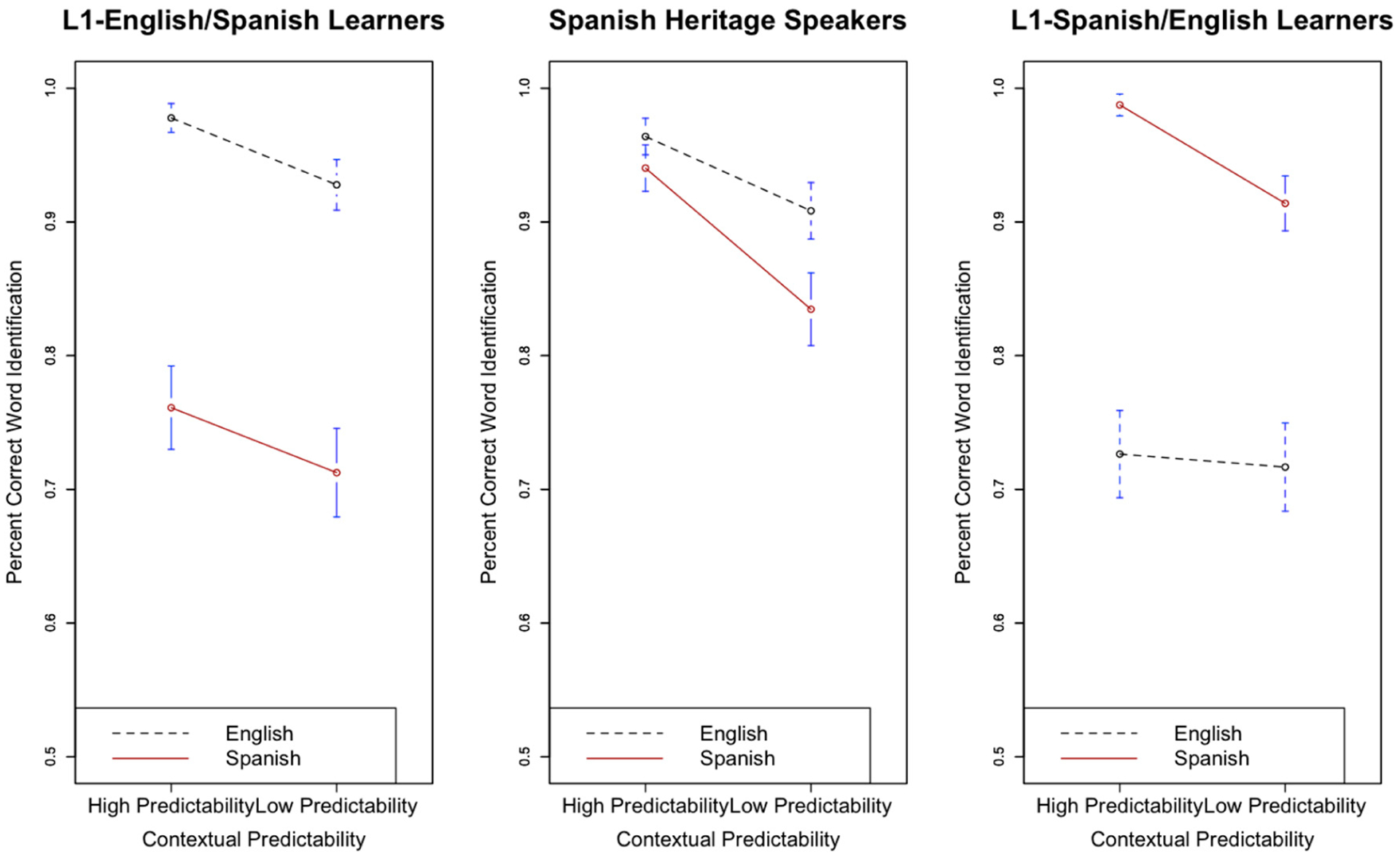
Effect of contextual (semantic) predictability on final word recognition in English and Spanish for L1-English/Spanish (L2) Learners (**left**), Spanish Heritage Speakers (**middle**), andL1-Spanish/English (L2) Learners (**right**). Bars indicate 95% confidence intervals around the means.

Figure 3 displays the effect of semantic predictability (high versus low) on English and Spanish speech-in-noise recognition for all three listener groups.

With respect to L1-E/SL versus SHS, there was a significant main effect of predictability (β = 0.8, s.e. β = 0.2, χ^2^(1) = 4.19, *p* < 0.05), indicating that highly semantically predictable contexts aided the recognition of final keywords. The interaction between listener group and predictability was significant (β = 0.61, s.e. β = 0.29, χ^2^(1) = 8.01, *p* < 0.05), such that SHS benefitted more from semantic predictability than L1-E/SL. Although the interaction between predictability and language was not significant (β = −0.45, s.e. β = 0.36, χ^2^(1) = 1.19, *p* > 0.05), the three-way interaction between listener group (L1-E/SL versus SHS), language, and predictability was significant (β = −1.29, s.e. β = 0.47, χ^2^(1) = 9.51, *p* < 0.05). This suggests that SHS and L1-E/SL did not equally benefit from predictability across both languages. While the two groups showed similar benefits from predictability in English, SHS showed a much stronger predictability benefit in Spanish than L1-E/SL. Consistent with Bradlow and Alexander (2007), there was an interaction between speech style and predictability (β = 0.79, s.e. β = 0.18, χ^2^(1) = 32.79, *p* < 0.05), indicating that listeners were aided more by clear speech in high predictability contexts.

With respect to L1-S/EL versus SHS, there was a significant main effect of predictability (β = 1.46, s.e. β = 0.22, χ^2^(1) = 14.53, *p* < 0.05) such that keywords were more accurately recognized when there was ample supporting context in the sentence that preceded them. While both the interaction between listener group and predictability (β = 0.04, s.e. β = 0.33, t = 0.14, χ^2^(1) = 1.13, *p* > 0.05) and language and predictability (β = −0.52, s.e. β = 0.45, χ^2^(1) = 1.11, *p* > 0.05) were not significant, there was a significant three-way interaction between predictability, language, and listener group (β = 2.6, s.e. β = 0.82, χ^2^(1) = 9.18, *p* < 0.05), suggesting that SHS and L1-S/EL did not equally benefit from predictability across both languages. That is, although both SHS and L1-S/EL benefitted from predictability in Spanish, SHS showed a much stronger predictability benefit in English compared to L1-S/EL. Again, consistent with Bradlow and Alexander, the interaction between style and predictability was significant (β = 1.09, s.e. β = 0.19, χ^2^(1) = 41.62, *p* < 0.05) such that clear speech more greatly aided word recognition in highly predictable contexts than in low predictability contexts.

### 3.3. Signal-to-Noise Ratio (SNR)

**Figure 4.**
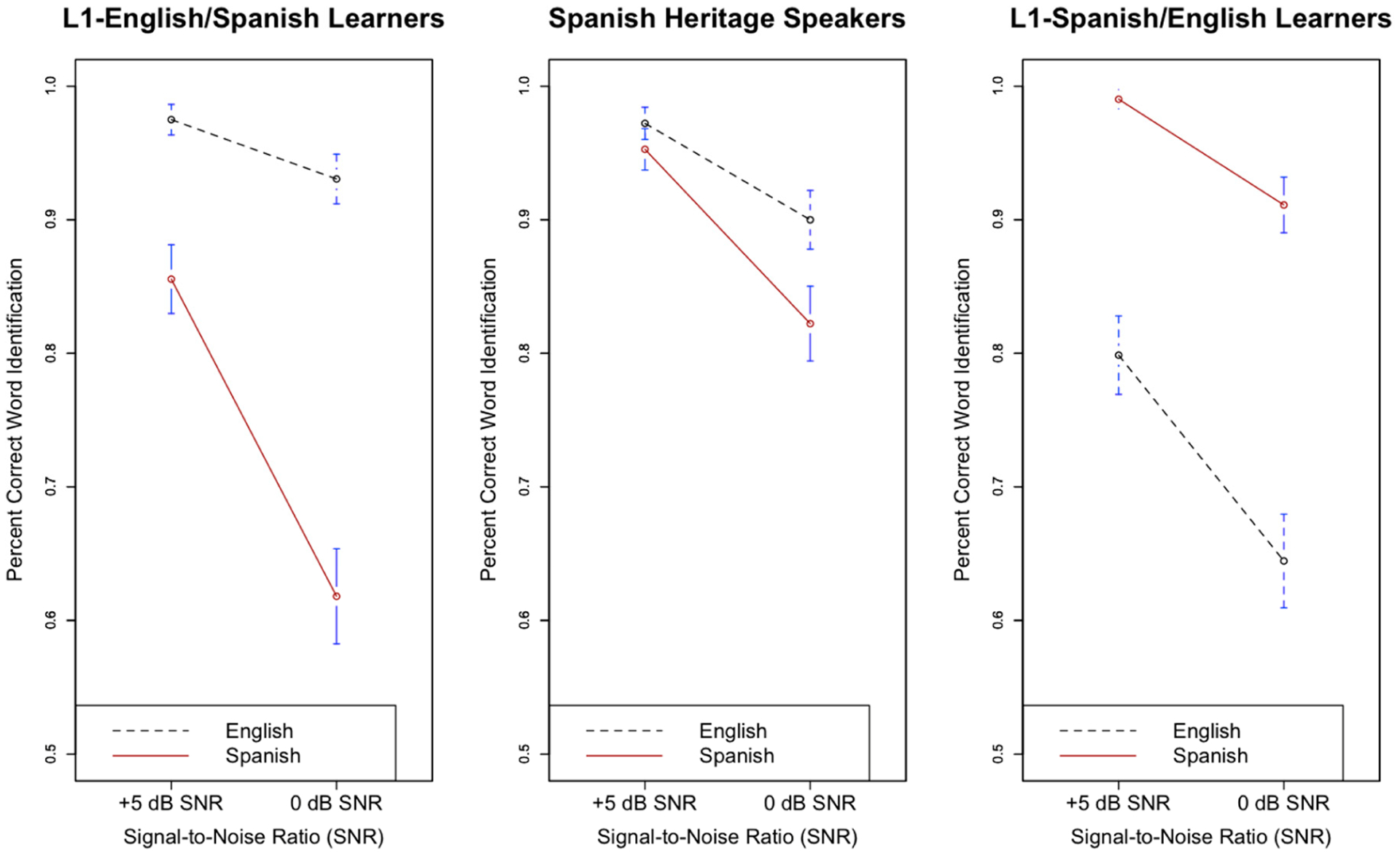
Effect of signal-to-noise ratio (SNR) on final word recognition in English and Spanish for L1-English/Spanish (L2) Learners (**left**), Spanish Heritage Speakers (**middle**), and L1-Spanish/English (L2) Learners (**right**). Bars indicate 95% confidence intervals around the means.

Figure 4 displays the effect of SNR (+5 db and 0 dB) on English and Spanish speech-in-noise recognition for all three listener groups.

With respect to L1-E/SL versus SHS, there was a significant main effect of SNR (β = 1.64, s.e. β = 0.13, χ^2^(1) = 52.7, *p* < 0.05), suggesting that final keyword recognition was improved in the favorable SNR condition, +5 dB. The interaction between SNR and language was significant (β = −0.64, s.e. β = 0.19, χ^2^(1) = 6.59, *p* < 0.05), indicating that both L1-E/SL and SHS were more resistant to noise in English than Spanish. The interaction between listener group and SNR was not significant (β = 0.25, s.e. β = 0.32, χ^2^(1) = 0.47, *p* > 0.05), nor was the three-way interaction between SNR, listener group (L1-E/SL versus SHS), language (β = 0.12, s.e. β = 0.49, χ^2^(1) = 0.049, *p* > 0.05), indicating that L1-E/SL and SHS were comparably affected by SNR manipulations in both languages. Both the interaction between style and SNR (β = −0.19, s.e. β = 0.18, χ^2^(1) = 0.01, *p* > 0.05) and the interaction between predictability and SNR (β = 0.09, s.e. β = 0.18, χ^2^(1) = 0.99, *p* > 0.05) were not significant.

With respect to L1-S/EL versus SHS, there was a significant main effect of signal-to-noise ratio (SNR) (β = 1.94, s.e. β = 0.14, χ^2^(1) = 52.8, *p* < 0.05) as final keyword recognition improved at the easier SNR (+5 dB). The three-way interaction between SNR, language, and listener group was significant (β = 1.53, s.e. β = 0.72, χ^2^(1) = 4.35, *p* < 0.05), suggesting that SHS and L1-S/EL were differentially affected by noise across both languages. While the interaction between listener group and SNR was not significant (β = −0.27, s.e. β = 0.34, χ^2^(1) = 0.01, *p* > 0.05), there was a significant interaction between language and SNR (β = −0.83, s.e. β = 0.28, t = −2.98, χ^2^(1) = 6.46, *p* < 0.05). Taken together, these results suggest that overall while SHS and L1-S/EL were similarly affected by noise, SHS were more resistant to the presence of noise in English compared to L1-S/EL. Furthermore, the interaction between style and SNR was not significant (β = −0.14, s.e. β = 0.19, χ^2^(1) = 0.21, *p* > 0.05). However, the interaction between predictability and SNR was significant (β= 0.39, s.e. β= 0.19, χ^2^(1) = 8.05, *p* < 0.05), indicating that highly predictable contexts aided keyword recognition in higher levels of noise.

## 4. Discussion

The goal of this study was to determine how the timing (i.e., “when”) and/or the amount (i.e., “how much”) of linguistic exposure affected speech learning outcomes in adults. Due to the de-confounding of these aspects of the language learning context—early versus extended language exposure—in switched-dominance bilinguals (L2 = dominant language; i.e., “heritage speakers”), we were able to investigate their independent influences on speech recognition accuracy under various enhancement/degradation conditions. Specifically, we compared recognition accuracy of sentence-final words in spoken sentences embedded in background noise by L2-dominant Spanish Heritage Speakers (SHS, L1-Spanish, L2-English) as compared to L1-dominant bilinguals in both languages. In the current study, we manipulated three factors that are known to either enhance or degrade recognition of sentence-final keywords: contextual predictability (Bradlow and Alexander 2007; Shi 2010), signal-to-noise ratio (Mayo et al. 1997; Shi 2010), and speech style (Bradlow and Bent 2002; Bradlow and Alexander 2007). These factors are known to differentially affect speech recognition by L1 and L2 listeners, with L2 listeners generally deriving a less marked benefit from each source of signal enhancement. As such, manipulating these factors provides a sensitive test of perceptual vulnerabilities found in bilingual speech recognition. Many previous studies on L1 and L2 speech perception (Mayo et al. 1997; Cutler et al. 2008; Shi 2010, etc.) have only shown results in one language (typically, the L2). However, the current study manipulates these factors in both languages in order to assess which, if either, of the SHS speaker’s two languages showed the perceptual vulnerabilities that are typical of the non-dominant L2.

One hypothesis is that if early exposure is critical for complete L1-like performance in a language (Mayo et al. 1997; Montrul 2005), heritage speakers would show better speech-in-noise recognition in both languages. Overall, the results showed that Spanish Heritage Speakers (SHS) were indeed more accurate than L1-English/Spanish learners (L1-E/SL) at final keyword recognition in Spanish under adverse listening conditions, establishing the benefit of early exposure for speech recognition in noise. Moreover, the results showed that in English, the dominant language for both of these groups, no differences were observed, establishing that both groups benefitted similarly from extended/continuous exposure to English. Additionally, we found that SHS speakers benefitted from contextual predictability in both languages; in contrast, Spanish and English learners failed to benefit from semantic predictability in their L2. These results support the early exposure hypothesis in that heritage speakers demonstrated dominant L1-like performance in certain aspects of both Spanish and English final word recognition due to their early acquisition of both English and Spanish.

An alternative hypothesis emphasizes the critical role of language dominance affecting language processing (Cutler et al. 1992; Montrul 2010), predicting vulnerabilities to emerge in the non-dominant language, regardless of the order in which the languages were acquired. We have support for this hypothesis from the two following results. First, while the SHS showed similar final word recognition accuracies in English compared to the L1-dominant English listeners (L1-E/SL), the SHS showed significantly lower final word recognition accuracy in Spanish compared to L1-dominant Spanish listeners (L1-S/EL). Furthermore, SHS only received a benefit of clear speech in English (their dominant L2) but not in Spanish (their non-dominant L1). These results are consistent with Cutler et al. (1992) and Kohnert et al. (1999) who found that when language proficiency and age of acquisition, respectively, were controlled for bilinguals demonstrated language processing difficulties in their non-dominant language. These results suggest that language dominance plays a critical role in bilingual speech processing, while early acquisition, although necessary, is not sufficient to provide resistance against all signal degradations. That is, when vulnerabilities arise for switched-dominance bilinguals, they are more likely to be found in the non-dominant language independent of the order in which the language was acquired.

With respect to non-dominant L2 learners (L1-English/Spanish learners (L1-E/SL) and L1-Spanish/English learners (L1-S/EL) in this study), these hypotheses do not make opposing predictions since the L2 for these bilinguals is both late-acquired and non-dominant. If early AoA (Mayo et al. 1997) and language dominance (Montrul 2010) are critical for speech-in-noise recognition, then non-dominant, late L2 learners are predicted to perform worse in their L2 compared to their L1. The effects of signal enhancements on L1-E/SL and L1-S/EL are consistent with these hypotheses’ predictions and several previous studies that highlight differences between L1 and L2 processing (Mayo et al. 1997; Bradlow and Bent 2002; Bradlow and Alexander 2007; Shi 2010), since both groups did not benefit as greatly from signal enhancements in their respective L2, non-dominant languages compared to L1 listeners. That is, Spanish and English L2 learners were more vulnerable to poorer SNRs and failed to extract salient information from contextual predictability and clear speech styles as effectively in their L2 as in their L1.

Why did the SHS fail to benefit from clear speech in Spanish despite benefitting in both Spanish and English from each of the other two enhancements tested in this study (SNR and predictability)? One possible explanation for why SHS did not receive a Spanish clear speech benefit is that the simultaneous Spanish-English bilingual used for stimuli recording did not produce a strong clear speech effect in Spanish. However, if this were true, then we would expect to see no benefit for L1-E/SL or L1-S/EL in Spanish clear speech. This is not the case (see Figure 2); in fact, the benefit is quite large, especially for L1-E/SL. Another possible explanation is that because SHS are not accustomed to receiving clear speech in family settings at home, they fail to benefit from acoustic redundancies and enhancements in the signal that clear speech can provide. Recall that SHS, by definition, are not educated in Spanish as a medium of instruction nor is Spanish the dominant language of US society and culture. Therefore, they are rarely exposed to hyper-articulated styles that generally come with pedagogical and formal speaking registers (Gutiérrez 1997). L1-E/SL, on the other hand, most likely receive the majority of their Spanish instruction in clear speech (Gilbert (1993), for example, has proposed clear speech as a tool for second language pedagogy). This pedagogical approach may have influenced Spanish learners by teaching them to benefit from acoustic correlates of clear speech in Spanish. It should be noted, however, that heritage speakers most likely received infant-directed Spanish (IDS), a type of hyper-articulated speech, from their parents and family (Polinsky and Kagan 2007). However, while IDS may contribute to heritage speakers learning the acoustic correlates of Spanish clear speech, it is restricted only to the first few years of life and does not extend into early childhood. L1-S/EL also unsurprisingly benefitted from clear speech in Spanish, suggesting that the lack of a clear speech benefit for SHS stems from their limited use of Spanish rather than a failure to effectively produce clear speech on the part of the talker used to record the stimuli.

## 5. Conclusions

The current study found that Spanish Heritage Speakers (SHS) who are dominant in their L2, English, demonstrated speech-in-noise recognition accuracy at similar levels to L1-dominant English listeners. However, speech recognition vulnerabilities related to signal degradation emerged for these SHS in their non-dominant L1, Spanish. Combined with generally better SHS than late L2 Spanish learner recognition accuracy of Spanish, these results suggest that while early exposure can afford substantial speech processing benefits, extended exposure and usage are necessary for robust L1-like speech-in-noise recognition. While the data from this study revealed subtle differences in non-dominant language speech recognition compared to their dominant language, it is critical to acknowledge that the bilingual language learning experience of switched dominance/heritage speakers affords obvious life-long benefits in terms of total language proficiency. In the present study, the sum of the English and Spanish cloze test scores for all three listener groups showed that the total language proficiencies of the Spanish Heritage Speakers exceeded that of both the L1-English/Spanish learners and the L1-Spanish/English learners, emphasizing the remarkable language asset of the heritage language learner experience.

## Footnotes

1 Grammar here refers to knowledge of morpho-syntax that is independent of speech production and/or perception as the majority of work on heritage speakers has focused on heritage-language morpho-syntax acquisition.

2 Here, L1 = dominant language; L2 = weaker, non-dominant language.

3 English proficiency was measured by a cloze test used in the lab of Guili Dussias at Pennsylvania State University (Kim 2008; Lim 2011). Spanish proficiency was measured by a cloze test modeled after the Dussias English cloze test, developed in the lab of Kiel Christianson at the University of Illinois (Gordon and Christianson 2010; Steinmeitz and Christianson Forthcoming).

4 Here, we use “clear” and “plain” as terms that reflect differences in intelligibility-related speech styles to differentiate “plain” from “conversational” speech. The term “plain” speech is not related to discourse-level conversational speech, as the stimuli used in this study were recorded from a prepared written script under monologue conditions.

## Appendix A

Stimuli used in study; Keywords are always sentence final

Spanish sentences: High Predictability

El esposo de la reina es el rey

Puedes depositar tu dinero en el banco

Quiere nadar en la piscina

El profesor trabaja en la oficina

Fue al cine para ver la película

A Susana le gusta escuchar la música

Ariel es popular y tiene muchos amigos

La química es una de las ciencias

Tiene una cámara y saca unas fotos

Es muy oscuro; necesito más luz

Raúl llora porque está muy triste

No es una mujer, es un hombre

Se cortó el dedo y hay mucha sangre

No escribas ni con una pluma ni un lápiz

Esta noche puedes conducir el coche

No me lo des hoy, dámelo mañana

El día que naciste es tu cumpleaños

La mano tiene cinco dedos

Cincuenta y dos semanas son un año

Escribe tu nombre y la fecha

Tengo sed y necesito una bebida

Limpia su coche porque está sucio

En el centro comercial fui de compras

Los coches están pasando por la calle

No hables cuando tienes comida en la boca

Jorge canta bien esa canción

Estudiaba mucho para el examen

Los católicos van a misa en la iglesia

La cocina tiene una mesa y unas sillas

Los estudiantes almuerzan en la cafetería

En casa le gusta ver la televisión

Hay treinta días en un mes

Va a dormir en su cama

El sol es una gran estrella

No entiendo y tengo una pregunta

Para graduarse, uno necesita tomar muchas clases

No te miento, digo la verdad

El verde es mi color favorito

Ganó la lotería y ahora es rico

Es hijo único y no tiene hermanos

No puedo ir porque tengo mucha tarea

Para tener aire fresco, abre la ventana

Necesito llamarte, dame tu número

Recibí una invitación para la fiesta

Estudia la bioquímica y es muy inteligente

El catolicismo es una religión

En los Estados Unidos se habla inglés

Hay sesenta minutos en una hora

No leí el libro y saqué una mala nota

El padre tiene dos hijos y una hija

Vas al gimnasio para hacer ejercicio

El novio besó a su novia

No tiene mucho dinero porque es pobre

Las vacas producen la leche

Vivo en una casa, no en un apartamento

Pedro quiere volar en un avión

Un diccionario contiene muchas palabras

La biblioteca tiene muchos libros

De noche hay luz de la luna

María tiene unos perros y unos gatos

Spanish sentences: Low Predictability

El niño vio el rey

La niña pensaba en el banco

Papá cree que es piscina

Mamá quiere decir oficina

Ellos discuten de la película

Sí, es música

El niño no pudo hablar de los amigos

Papá pensaba en ciencias

La niña vio las fotos

Mamá cree que es luz

Ellos quieren decir triste

El niño no pudo hablar del hombre

Sí, es sangre

La niña discute del lápiz

Papá discute del coche

Mamá cree que es mañana

Ellos no pudieron hablar del cumpleaños

Sí, son dedos

El niño pensaba en el año

La niña quiere decir fecha

Mamá vio la bebida

Papá quiere decir sucio

Ellos pensaban en compras

Sí, es calle

Mamá discute de la boca

El niño vio la canción

La niña cree que es examen

Papá no pudo hablar de la iglesia

Mamá vio las sillas

Ellos pensaban en la cafetería

El niño cree que es televisión

La niña quiere decir mes

Papá discute del cama

Sí, es estrella

Mamá no pudo hablar de la pregunta

Ello vieron las clases

El niño pensaba en la verdad

La niña quiere decir favorito

Mamá cree que es rico

Papá discute de los hermanos

Sí, es tarea

Ellos no pudieron hablar de la ventana

Mamá vio el número

Papá pensaba en la fiesta

Ellos creen que es inteligente

Sí, es religión

El niño quiere decir inglés

La niña discute de la hora

Mamá no pudo hablar de la nota

Sí, es hija

La niña vio el ejercicio

Mamá pensaba en la novia

Papá cree que es pobre

Ellos quieren decir leche

El niño discute del apartamento

La niña no pudo hablar del avión

Papá vio las palabras

Ellos pensaban en libros

El niño cree que es la luna

La niña quiere decir gatos

English sentences: High Predictability

The meat from a pig is called pork

For dessert he had apple pie

Sugar tastes very sweet

The color of a lemon is yellow

My clock was wrong so I got to school late

In spring, the plants are full of green leaves

A bicycle has two wheels

She made the bed with clean sheets

The sport shirt has short sleeves

He washed his hands with soap and water

The child dropped the dish and it broke

The bread was made from whole wheat

The opposite of hot is cold

A wristwatch is used to tell the time

The war plane dropped a bomb

She cut the cake with a knife

A chair has four legs

Cut the meat into small pieces

The team was trained by their coach

The lady wears earrings in her ears

People wear shoes on their feet

When sheep graze in a field, they eat grass

A rose is a type of flower

Football is a dangerous sport

The heavy rains caused a flood

Bob wore a watch on his wrist

Monday is the first day of the week

The pan that was just in the oven is very hot

Rain falls from clouds in the sky

The boy laughed because the joke was very funny

To cool her drink, she added a few cubes of ice

A quarter is worth twenty-five cents

An orange is a type of fruit

People wear scarves around their necks

I wrote my name on a piece of paper

For your birthday I baked a cake

Birds build their nests in trees

My parents, sister and I are a family

The good boy is helping his mother and father

People wear gloves on their hands

A book tells a story

A pigeon is a kind of bird

The sick woman went to see a doctor

The lady uses a hairbrush to brush her hair

At breakfast he drank some orange juice

Last night, they had beef for dinner

A racecar can go very fast

Many people like to start the day with a cup of coffee

He brought the book to school from home

I wear my hat on my head

Red and green are colors

The stars come out at night

February has twenty-eight days

The picture is hung high on the bedroom wall

We heard the ticking of the clock

She laid the meal on the table

She looked at herself in her mirror

Elephants are big animals

After my bath, I dried off with a towel

In the morning it gets light, and in the evening it gets dark

English sentences: Low predictability

Dad looked at the pork

Mom talked about the pie

We think that it is sweet

Mom thinks that it is yellow

He thinks that it is late

She talked about the leaves

He read about the wheels

Dad talked about the sheets

He looked at the sleeves

We talked about the water

We heard that it broke

Dad pointed at the wheat

She thinks that it is cold

This is her favorite time

Dad talked about the bomb

Mom read about the knife

She looked at her legs

There are many pieces

We read about the coach

She pointed at his ears

Mom looked at her feet

Dad pointed at the grass

She read about the flower

This is her favorite sport

He read about the flood

He looked at her wrist

This is her favorite week

Mom thinks that it is hot

Dad read about the sky

Dad thinks that it is funny

He talked about the ice

He pointed at the cents

He pointed at the fruit

She talked about their necks

We talked about the paper

This is her favorite cake

He read about the trees

We read about the family

Mom pointed at his father

She looked at her hands

We looked at the story

We pointed at the bird

Mom talked about the doctor

He pointed at his hair

Mom looked at the juice

He talked about the dinner

She thinks that it is fast

Mom pointed at the coffee

She pointed at the home

She pointed at her head

Mom read about the colors

This is her favorite night

There are many days

We pointed at the wall

She looked at the clock

Dad read about the table

We looked at the mirror

He pointed at the animals

Dad looked at the towel

Dad thinks that it is dark
